# Dietary Apilarnil Supplementation Modulates Erythrocyte Redox Enzyme Activities in Rats Subjected to Repeated Swimming Exercise

**DOI:** 10.3390/metabo16100791

**Published:** 2026-10-19

**Authors:** Halil Şimşek, Fatih Çakar

**Affiliations:** Vocational School of Health Services, Bingöl University, 12000 Bingöl, Türkiye

**Keywords:** apilarnil, erythrocyte, glucose-6-phosphate dehydrogenase, NADPH, oxidative stress, swimming exercise

## Abstract

**Highlights:**

**What are the main findings?**

- Repeated swimming with a terminal exhaustive challenge, but not acute exhaustive swimming alone, was associated with higher erythrocyte redox enzyme activities.
- Apilarnil was associated with lower GR and TrxR (both doses) and GST (higher dose).

**What are the implications of the main findings?**

- Apilarnil appears to modulate thiol-related rather than NADPH-generating erythrocyte enzymes.
- The findings indicate enzymatic modulation, not proven antioxidant protection; direct redox markers are needed.

**Abstract:**

Background: Exercise-induced reactive oxygen species (ROS) participate in both oxidative stress and physiological adaptation. Erythrocytes rely on NADPH-generating and thiol-related antioxidant systems to maintain redox homeostasis during exercise. Apilarnil, a drone brood-derived bee product containing diverse bioactive compounds, may influence redox-related enzymatic responses; however, its effects on erythrocyte NADPH-dependent and thiol-related enzyme activities during repeated exercise remain unclear. Methods: Forty adult male Wistar rats were randomly assigned to five groups (n = 8/group): control (CN), acute exhaustive swimming (AES), short-term repeated swimming with vehicle (ST), repeated swimming plus apilarnil 0.2 g·kg^−1^·day^−1^ (ST + AP0.2), and repeated swimming plus apilarnil 0.4 g·kg^−1^·day^−1^ (ST + AP0.4). Repeated-swimming groups swam for 30 min/day for 14 consecutive days. Apilarnil was administered orally throughout the intervention. On Day 15, the AES and repeated-swimming groups underwent a terminal exhaustive swimming challenge. Erythrocyte G6PD, 6PGD, GST, GR, and TrxR activities were determined spectrophotometrically. Results: Acute exhaustive swimming alone did not significantly alter erythrocyte enzyme activities. In contrast, repeated swimming exposure followed by the terminal exhaustive challenge was associated with higher G6PD, 6PGD, GST, GR, and TrxR activities compared with sedentary controls. Compared with ST, the higher apilarnil dose was associated with lower GST activity, and both doses were associated with lower GR and TrxR activities. G6PD and 6PGD activities did not differ significantly between apilarnil-treated and ST groups. Conclusions: Within the context of short-term repeated swimming, apilarnil-supplemented rats showed lower activities of selected erythrocyte redox enzymes than vehicle-treated rats. Because direct oxidative stress markers, hemolysis-related outcomes, and upstream molecular pathways were not assessed, these findings should be interpreted as evidence of enzymatic modulation rather than definitive evidence of oxidative protection or enhanced antioxidant adaptation.

## 1. Introduction

Strenuous exercise increases oxygen consumption and promotes reactive oxygen species (ROS) generation through mitochondrial, enzymatic, inflammatory, and hemoglobin-related redox reactions [1]. Although excessive ROS production may contribute to oxidative damage, transient and controlled ROS signaling is also required for physiological adaptation to exercise [2]. This dual role forms the basis of exercise-induced redox hormesis, whereby repeated exercise exposure may strengthen endogenous antioxidant defense rather than merely produce oxidative injury [3,4,5].

Erythrocytes are particularly relevant for evaluating systemic redox responses to exercise [6]. They are continuously exposed to high oxygen tension and hemoglobin auto-oxidation, yet mature erythrocytes lack nuclei and mitochondria. Therefore, they depend largely on NADPH-generating and thiol-based antioxidant systems to preserve redox balance and membrane integrity [7,8,9]. The pentose phosphate pathway (PPP), mainly through glucose-6-phosphate dehydrogenase (G6PD) and 6-phosphogluconate dehydrogenase (6PGD), is the principal source of NADPH in erythrocytes [10]. NADPH, in turn, supports glutathione reductase (GR) and thioredoxin reductase (TrxR), while glutathione S-transferase (GST) contributes to detoxification of oxidized and electrophilic products [11,12,13]. Thus, changes in these enzyme activities may provide useful information on erythrocyte redox responses during acute and repeated exercise exposure.

The nutritional modulation of exercise-induced oxidative stress remains controversial. Classical antioxidant supplements such as vitamins C and E have been widely studied, but high-dose antioxidant supplementation may blunt some ROS-dependent training adaptations, a phenomenon described as the “antioxidant paradox” [14,15,16]. In contrast, polyphenol-rich and multi-component natural products may act not only as direct radical scavengers but also as modulators of redox-sensitive signaling, inflammatory pathways, and endogenous antioxidant enzymes [17,18,19,20]. Such effects have been investigated not only in exercise models but also in oxidative stress-related pathological conditions, including metabolic and inflammatory disorders. Therefore, evaluating whether a bioactive supplement suppresses, preserves, or modulates exercise-associated redox responses is essential.

Apilarnil, a drone brood-derived bee product, is a complex natural matrix containing amino acids, peptides, lipids, vitamins, phenolic acids, and flavonoids. Compared with other apiproducts such as honey, propolis, and royal jelly, apilarnil has been less extensively studied in exercise physiology and erythrocyte redox biology. Previous experimental studies suggest that apilarnil may exert antioxidant and anti-inflammatory effects in models of systemic injury [21,22]. Its phenolic and flavonoid constituents may provide a plausible biological context for potential redox-related effects; however, the individual contribution, bioavailability, and mechanisms of action of these compounds were not investigated in the present study. Therefore, the effects observed here should be considered as responses to the apilarnil matrix rather than effects attributable to any single constituent.

Despite increasing interest in bee-derived products and exercise-related oxidative stress, the effects of apilarnil on erythrocyte NADPH-dependent and thiol-related enzyme activities remain unclear. In particular, it is not known whether a single acute exhaustive swimming challenge produces measurable changes in these enzymes, whether repeated swimming exposure followed by the same terminal challenge is associated with a different enzymatic response, or whether apilarnil supplementation during the repeated exercise period modifies these responses.

Therefore, this experimental study aimed to evaluate erythrocyte G6PD, 6PGD, GST, GR, and TrxR activities in rats exposed to acute exhaustive swimming or repeated swimming exercise followed by a terminal exhaustive challenge, and to determine whether apilarnil supplementation during the repeated exercise period modifies these enzyme activity responses. We hypothesized that repeated swimming exposure followed by a terminal exhaustive challenge would be associated with different erythrocyte PPP-associated and thiol-related enzyme activity responses compared with acute exhaustive swimming alone, and that apilarnil supplementation during the repeated exercise period would modify selected enzyme activity responses.

## 2. Materials and Methods

### 2.1. Animals and Ethical Approval

This experimental study was conducted using 40 adult male Wistar albino rats obtained from the Experimental Animals Production and Research Unit of Bingöl University, Türkiye. Animals were housed under standard laboratory conditions at 22 ± 2 °C, 50–60% relative humidity, and a 12:12 h light/dark cycle, with free access to standard rat chow and water. Only male rats were included in this initial study. We recognize, however, that sex is a relevant biological variable in exercise physiology and redox biology rather than merely a source of variability.

The experimental protocol was reviewed and approved by the Animal Experiments Local Ethics Committee of Bingöl University (Approval No: E-85680299-020-2600009350/10.02.2026). All experimental procedures were conducted in accordance with national and international principles for the care and use of laboratory animals and were reported in accordance with the ARRIVE guidelines [23]. All efforts were made to minimize animal suffering and to use the minimum number of animals required to obtain interpretable experimental data.

### 2.2. Experimental Design

The study was designed to compare basal erythrocyte enzyme activity, the response to a single terminal acute exhaustive swimming challenge, the effect of short-term repeated swimming exposure followed by the same terminal challenge, and the effect of apilarnil supplementation during short-term repeated swimming exposure. The experimental design is summarized in Table 1.

Forty rats were randomly allocated to five groups (n = 8 per group) by simple randomization using a lottery (drawing lots) procedure. The investigator performing the biochemical analyses was blinded to group allocation. The number of animals per group was determined by considering previous experimental swimming and oxidative stress-related biochemical studies using comparable rat models, the need for sufficient biological replication in enzyme activity measurements, and the ethical principle of reduction in animal research.

Control group (CN): Rats in the CN group served as basal sedentary controls. They did not receive apilarnil, did not undergo oral gavage, did not perform repeated swimming, and were not exposed to the terminal acute exhaustive swimming challenge.

Acute exhaustive swimming group (AES): Rats in the AES group received physiological saline by oral gavage at 0.5 mL/day for 14 days. They did not receive apilarnil supplementation and did not undergo the 14-day repeated swimming protocol. At the end of the experimental period, they were exposed only to a single terminal acute exhaustive swimming session.

Repeated swimming group (ST): Rats in the ST group performed swimming exercise for 30 min/day for 14 consecutive days. They received physiological saline by oral gavage at 0.5 mL/day during the intervention period and were exposed to the terminal acute exhaustive swimming challenge at the end of the experiment.

Repeated swimming plus low-dose apilarnil group (ST + AP0.2): Rats in the ST + AP0.2 group followed the same repeated swimming and terminal acute exhaustive swimming protocol as the ST group. In addition, they received apilarnil by oral gavage at a dose of 0.2 g·kg^−1^·day^−1^ during the 14-day intervention period.

Repeated swimming plus high-dose apilarnil group (ST + AP0.4): Rats in the ST + AP0.4 group followed the same repeated swimming and terminal acute exhaustive swimming protocol as the ST group. In addition, they received apilarnil by oral gavage at a dose of 0.4 g·kg^−1^·day^−1^ during the 14-day intervention period. 

Thus, the AES group was used to distinguish the effect of a single acute exhaustive swimming challenge from the effect of repeated swimming exposure, whereas the apilarnil-treated groups were used to evaluate whether erythrocyte enzyme activities differed between vehicle- and apilarnil-treated animals within the repeated-swimming context at two doses.

### 2.3. Rationale for Apilarnil Administration During Repeated Swimming 

Apilarnil was administered during the 14-day swimming period to model repeated nutritional exposure accompanying repeated exercise rather than a single-dose acute intervention. The supplementation protocol was therefore intended to examine whether apilarnil modified erythrocyte enzyme activity responses associated with repeated swimming exposure followed by a terminal exhaustive challenge. The study was not designed to determine the independent effects of apilarnil under sedentary conditions.

### 2.4. Apilarnil Preparation and Administration

Apilarnil (drone brood homogenate) was purchased from Harşena Arı Ürünleri (Amasya, Türkiye; registration no. DSL 590; batch no. 02; expiration date 22 October 2026). The term “apilarnil” is used here as the generic name of the product, not as a registered trademark of the manufacturer. All animals received material from this single batch to avoid batch-to-batch variability.

According to the production procedure, the product was obtained from *Apis mellifera* L. drone larvae harvested from comb cells at 7–9 days of development. The larvae were homogenized, frozen at −80 °C for 24 h, and lyophilized under vacuum for 48 h. The lyophilized material was ground into a fine powder and stored in sterile, airtight glass bottles at −20 °C, protected from light, until use [24]. Dosing suspensions were prepared freshly each day by suspending the lyophilized powder in physiological saline and were administered by oral gavage in a volume of 0.5 mL. Doses (0.2 and 0.4 g·kg^−1^·day^−1^) refer to the lyophilized powder.

The phenolic composition of the same product was previously determined by liquid chromatography–tandem mass spectrometry (LC-MS/MS) [23]. Briefly, the powder was extracted with 75% ethanol–water (1:10, *w*/*v*) in the dark at room temperature, centrifuged at 10,000 rpm for 10 min, and concentrated under vacuum at 40 °C. The extract was analyzed on a Shimadzu Nexera UHPLC system coupled to an LCMS-8040 tandem mass spectrometer (spectrometer (both from Shimadzu Corporation, Kyoto, Japan), operated with electrospray ionization in positive and negative modes and multiple reaction monitoring, using a validated method for the quantification of 53 phytochemicals [25]. Thirty of these compounds were detected. Phenolic acids predominated, mainly quinic acid (29.86 mg/g extract), p-coumaric acid (6.08 mg/g), 4-hydroxybenzoic acid (4.40 mg/g), and caffeic acid (3.80 mg/g), followed by fumaric (1.57 mg/g), gentisic (1.51 mg/g), and protocatechuic acids (1.23 mg/g). The main flavonoids were quercetin (3.02 mg/g), acacetin (2.19 mg/g), kaempferol (2.13 mg/g), genistein (1.19 mg/g), and chrysin (0.88 mg/g); lower amounts of catechin, quercitrin, luteolin, and apigenin were also present. These values are expressed per gram of ethanolic extract rather than per gram of the lyophilized powder administered to the animals.

### 2.5. Swimming Acclimation and Exercise Protocol

Before the experimental protocol, rats assigned to swimming procedures underwent water acclimation to reduce novelty-related stress during subsequent exercise exposure. Swimming procedures were performed during the light phase between 09:00 and 12:00 to minimize circadian variability in physiological and biochemical responses. All swimming sessions were conducted in a Morris-type cylindrical tank with a diameter of 130 cm and a water depth of 50 cm. Water temperature was maintained at 31.0 ± 1.0 °C throughout the procedures.

Rats in the ST, ST + AP0.2, and ST + AP0.4 groups performed swimming exercise for 30 min/day for 14 consecutive days. The AES group did not participate in the repeated swimming protocol. On the day after completion of the 14-day intervention period, AES, ST, ST + AP0.2, and ST + AP0.4 groups were subjected to the terminal acute exhaustive swimming challenge. No additional external load was attached to the animals during the exhaustive swimming session. Exhaustion was defined as the inability of the rat to rise to the water surface for breathing for 10 s despite gentle stimulation. Gentle stimulation consisted of mild agitation of the water near the animal, without physical contact, repeated at 2–3 s intervals while the animal remained below the surface. The same stimulation procedure was applied to all animals in the AES, ST, ST + AP0.2, and ST + AP0.4 groups, and exhaustion was assessed by the same investigator for all animals. When this criterion was met, the animal was immediately removed from the water and dried [26]. No external workload was applied during the repeated swimming sessions, and exercise intensity was not individually quantified using blood lactate, indirect calorimetry, or other physiological threshold measurements. Given the short duration (14 days), the absence of external loading, and the lack of physiological quantification of intensity, this protocol is referred to as short-term repeated swimming exposure rather than conventional endurance training.

### 2.6. Blood Sampling and Euthanasia

Blood samples were collected 1 h after the end of the terminal swimming challenge, in line with previous studies in which blood and tissues were collected 1 h after exhaustive swimming in rats [27]; CN animals were sampled under the same conditions. At this time point, animals were deeply anesthetized with xylazine 10 mg·kg^−1^ and ketamine 100 mg·kg^−1^. Blood samples were collected from the abdominal vein into EDTA-containing tubes to prevent coagulation. Following blood collection under deep anesthesia, animals were euthanized by cervical dislocation. All procedures were performed by trained personnel to minimize pain and distress.

### 2.7. Erythrocyte Isolation and Hemolysate Preparation

Blood samples were centrifuged at 3000 rpm for 10 min at +4 °C. The plasma layer was carefully removed, and the erythrocyte pellet was washed three times with ice-cold isotonic saline solution (0.9% NaCl) to reduce residual plasma proteins and leukocyte contamination. After the final wash, erythrocytes were resuspended in 0.16 M KCl solution and lysed by the addition of distilled water. The hemolysate was then centrifuged at 10,000× *g* for 30 min at +4 °C to remove membrane debris and insoluble material. The clarified supernatant was collected and stored at −80 °C until biochemical analyses were performed.

### 2.8. Erythrocyte Enzyme Activity Assays

Erythrocyte enzyme activities related to NADPH generation and thiol-dependent antioxidant defense were measured spectrophotometrically in hemolysates using established manual methods. The analyzed enzymes were glucose-6-phosphate dehydrogenase (G6PD) and 6-phosphogluconate dehydrogenase (6PGD) as pentose phosphate pathway enzymes, and glutathione S-transferase (GST), glutathione reductase (GR), and thioredoxin reductase (TrxR) as antioxidant defense-related enzymes.

All enzyme activities were measured spectrophotometrically at 30 ℃. 

G6PD and 6PGD activities were determined according to Beutler [28] by measuring the rate of NADPH formation at 340 nm, using glucose-6-phosphate and 6-phosphogluconate as substrates, respectively, in Tris-HCl buffer (pH 8.0) containing NADP^+^ and MgCl_2_.

GR activity was measured according to Carlberg and Mannervik [29] by monitoring the decrease in absorbance at 340 nm due to NADPH oxidation during the reduction of oxidized glutathione (GSSG) in potassium phosphate buffer (pH 7.0). 

GST activity was determined according to Habig and Jakoby [30] by monitoring the formation of the conjugate between reduced glutathione (GSH) and 1-chloro-2,4-dinitrobenzene (CDNB) at 340 nm in potassium phosphate buffer (pH 6.5). 

TrxR activity was measured according to Holmgren and Björnstedt [31] by monitoring the NADPH-dependent reduction of 5,5′-dithiobis(2-nitrobenzoic acid) (DTNB) at 412 nm in potassium phosphate buffer (pH 7.0). One enzyme unit was defined as the amount of enzyme catalyzing the conversion of 1 µmol of substrate per minute under the assay conditions.

All hemolysate samples were prepared and analyzed using identical processing, storage, and assay conditions to support valid within-study comparisons among experimental groups. Since commercial kits were not used, the enzyme activity measurements were based on the cited spectrophotometric procedures and were performed under standardized laboratory conditions.

### 2.9. Protein Determination and Normalization of Enzyme Activity

Total protein concentration in erythrocyte hemolysates was determined using the Bradford protein assay [32]. A standard calibration curve was prepared using bovine serum albumin as the reference protein. Enzyme activities were normalized to total hemolysate protein concentration and expressed as enzyme units per milligram protein (EU/mg protein). This normalization approach was applied consistently across all samples and groups. Total hemolysate protein was used as the denominator because hemoglobin constitutes approximately 97–98% of the soluble protein content of erythrocytes, as shown in a human erythrocyte proteome study, so protein-based normalization closely approximates hemoglobin-based normalization [33]. The same Bradford-based denominator was applied uniformly to all samples.

### 2.10. Statistical Analysis

Statistical analyses were performed using GraphPad Prism version 10.6.1 (GraphPad Software, Boston, MA, USA). The normality of data distribution was evaluated using the Shapiro–Wilk test, and homogeneity of variance was assessed using the Brown–Forsythe and Bartlett tests. When neither test indicated heterogeneity of variance (GST, GR), differences among the five experimental groups were analyzed using ordinary one-way analysis of variance (ANOVA) followed by Tukey’s multiple comparisons test. When either test indicated heterogeneous variances (*p* < 0.05; G6PD, 6PGD, TrxR), Welch’s ANOVA followed by Dunnett’s T3 multiple comparisons test was used. ANOVA results are reported as F (ordinary ANOVA) or W (Welch’s ANOVA) statistics with degrees of freedom and *p* values. Effect sizes are expressed as eta squared (η^2^) and, for ordinary ANOVA, omega squared (ω^2^), calculated from the sums of squares of the ordinary one-way ANOVA. For key pairwise comparisons, mean differences with 95% confidence intervals (CIs) are reported.

Because a formal a priori power analysis was not performed, a sensitivity power analysis was conducted. With n = 8 per group (N = 40) and five groups, the study had 80% power at α = 0.05 to detect effects of f ≥ 0.58 (η^2^ ≥ 0.25) in the one-way ANOVA. For pairwise comparisons, using a Bonferroni-adjusted α for 10 comparisons as a conservative approximation, effects of approximately Cohen’s d ≥ 2.1 would be detectable. The study was therefore powered to detect only large effects, and smaller between-group differences may have remained undetected.

Data are presented as mean ± standard deviation (SD), with individual values shown. A *p* value of <0.05 was considered statistically significant. For graphical presentation, group differences were indicated using letter annotations: groups sharing the same letter were not significantly different from one another, whereas groups with different letters were significantly different at *p* < 0.05. A non-significant difference was not interpreted as evidence of equivalence or normalization.

## 3. Results

### 3.1. Erythrocyte G6PD Activity

**Figure 1:**
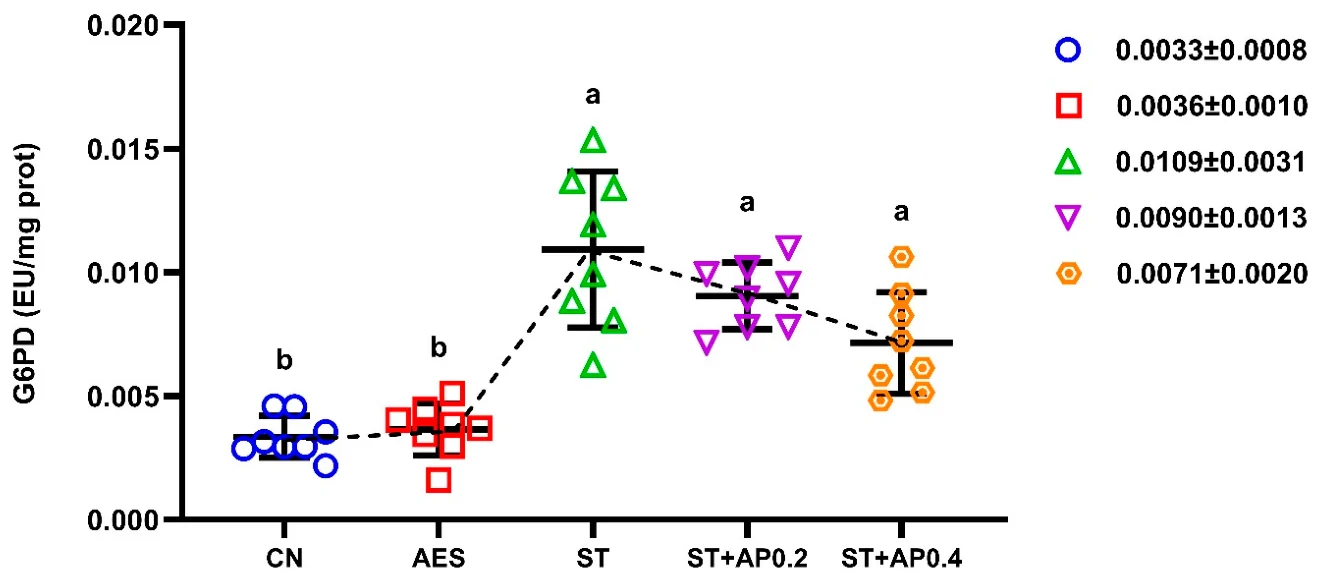
Erythrocyte G6PD activity across experimental groups. Data are presented as mean ± SD (n = 8 per group). Groups sharing the same letter are not significantly different, whereas groups with different letters differ significantly at *p* < 0.05 according to Welch’s ANOVA followed by Dunnett’s T3 multiple comparisons test. G6PD, glucose-6-phosphate dehydrogenase; CN, control; AES, acute exhaustive swimming; ST, short-term repeated swimming (vehicle-treated); ST + AP0.2 and ST + AP0.4, short-term repeated swimming plus apilarnil at 0.2 and 0.4 g·kg^−1^·day^−1^, respectively.

G6PD activity differed significantly among groups (Welch’s ANOVA: W(4.00, 16.93) = 34.12, *p* < 0.001; η^2^ = 0.74). Erythrocyte G6PD activity did not differ significantly between the CN and AES groups (*p* = 0.999). G6PD activity was significantly higher in the ST group than in both CN and AES groups (*p* = 0.002 and *p* = 0.001, respectively). Mean G6PD activity was numerically lower in the ST + AP0.2 and ST + AP0.4 groups than in the ST group; however, neither difference reached statistical significance (ST vs. ST + AP0.2: mean difference 0.0019 EU/mg protein, 95% CI −0.0024 to 0.0062, *p* = 0.72; ST vs. ST + AP0.4: mean difference 0.0038 EU/mg protein, 95% CI −0.0007 to 0.0082, *p* = 0.12). Both apilarnil-treated groups remained significantly higher than CN and AES (*p* < 0.05) (Figure 1).

### 3.2. Erythrocyte 6PGD Activity

**Figure 2:**
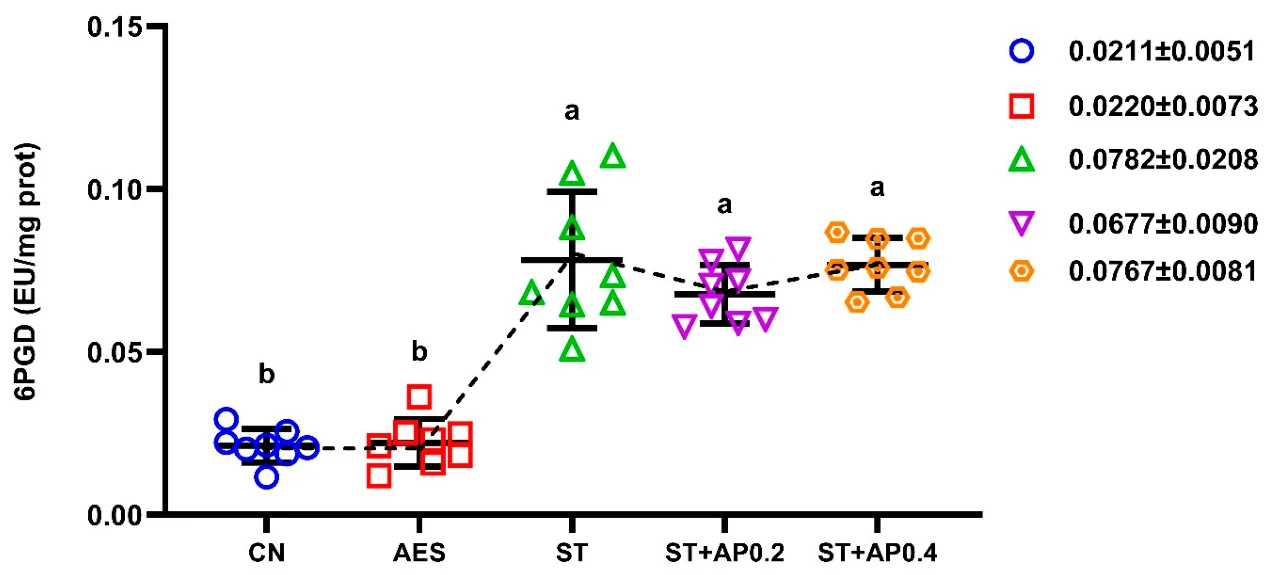
Erythrocyte 6PGD activity across experimental groups. Data are presented as mean ± SD (n = 8 per group). Groups sharing the same letter are not significantly different, whereas groups with different letters differ significantly at *p* < 0.05 according to Welch’s ANOVA followed by Dunnett’s T3 multiple comparisons test. 6PGD, 6-phosphogluconate dehydrogenase; CN, control; AES, acute exhaustive swimming; ST, short-term repeated swimming (vehicle-treated); ST + AP0.2 and ST + AP0.4, short-term repeated swimming plus apilarnil at 0.2 and 0.4 g·kg^−1^·day^−1^, respectively.

6PGD activity differed significantly among groups (Welch’s ANOVA: W(4.00, 17.02) = 93.54, *p* < 0.001; η^2^ = 0.85). Erythrocyte 6PGD activity did not differ significantly between the CN and AES groups (*p* > 0.999). 6PGD activity was significantly higher in the ST, ST + AP0.2, and ST + AP0.4 groups than in both CN and AES groups (all *p* < 0.001). No significant differences were observed among the ST, ST + AP0.2, and ST + AP0.4 groups (ST vs. ST + AP0.2: mean difference 0.0105 EU/mg protein, 95% CI −0.0174 to 0.0384, *p* = 0.86; ST vs. ST + AP0.4: mean difference 0.0015 EU/mg protein, 95% CI −0.0267 to 0.0296, *p* > 0.999) (Figure 2).

### 3.3. Erythrocyte GST Activity

**Figure 3:**
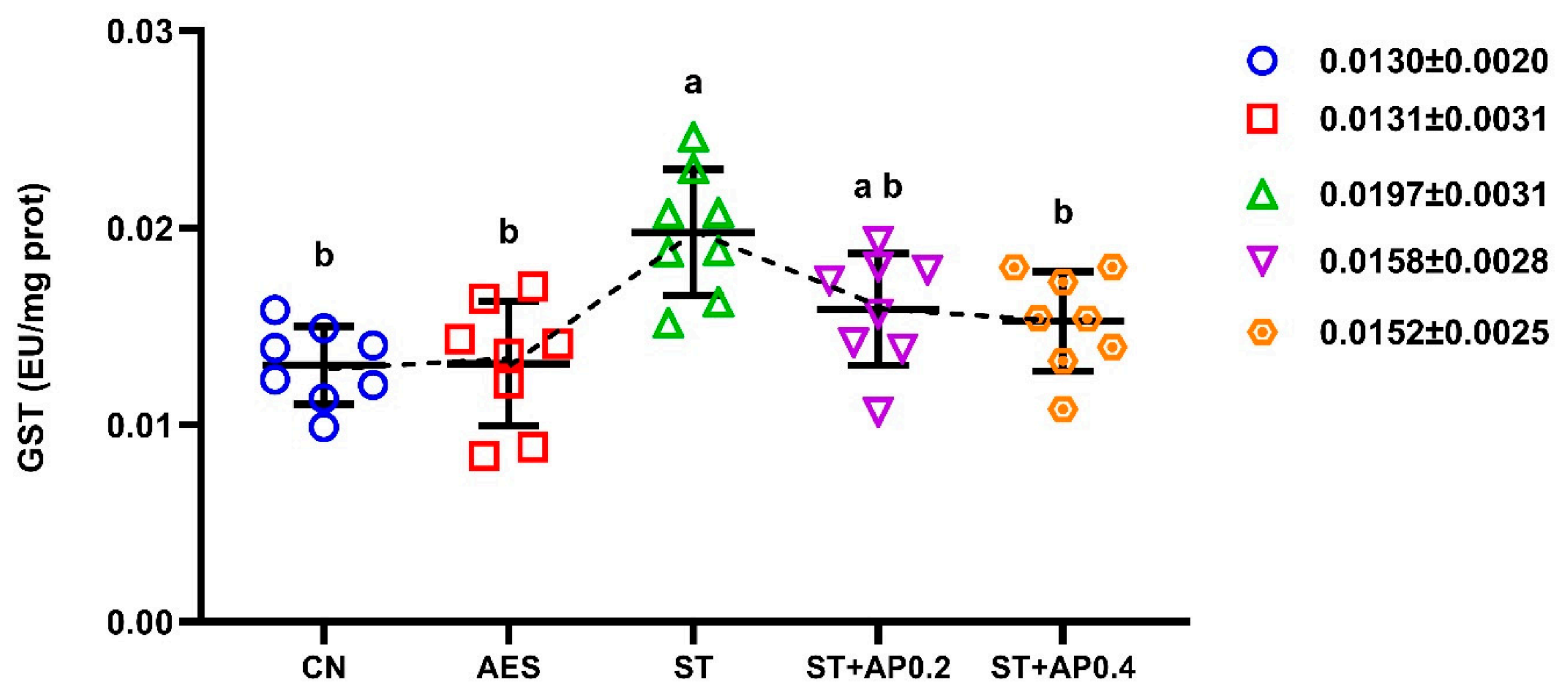
Erythrocyte GST activity across experimental groups. Data are presented as mean ± SD (n = 8 per group). Groups sharing the same letter are not significantly different, whereas groups with different letters differ significantly at *p* < 0.05 according to one-way ANOVA followed by Tukey’s multiple comparisons test. GST, glutathione S-transferase. CN, control; AES, acute exhaustive swimming; ST, short-term repeated swimming (vehicle-treated); ST + AP0.2 and ST + AP0.4, short-term repeated swimming plus apilarnil at 0.2 and 0.4 g·kg^−1^·day^−1^, respectively.

GST activity differed significantly among groups (F(4, 35) = 7.80, *p* < 0.001, η^2^ = 0.47, ω^2^ = 0.40). Erythrocyte GST activity did not differ significantly between the CN and AES groups (*p* > 0.999). GST activity was significantly higher in the ST group than in both CN and AES groups (*p* < 0.001 for both). Compared with ST, GST activity was significantly lower in the ST + AP0.4 group (mean difference 4.51 × 10^−3^ EU/mg protein, 95% CI 0.51 × 10^−3^ to 8.50 × 10^−3^, *p* = 0.02), whereas the difference between ST and ST + AP0.2 did not reach statistical significance (mean difference 3.91 × 10^−3^ EU/mg protein, 95% CI −0.09 × 10^−3^ to 7.90 × 10^−3^, *p* = 0.058). GST activity in the ST + AP0.2 and ST + AP0.4 groups was not statistically distinguishable from CN or AES under the current sample size (*p* > 0.05), which should not be interpreted as equivalence to control values. No significant difference was observed between the two apilarnil doses (*p* = 0.99) (Figure 3).

### 3.4. Erythrocyte GR Activity

**Figure 4:**
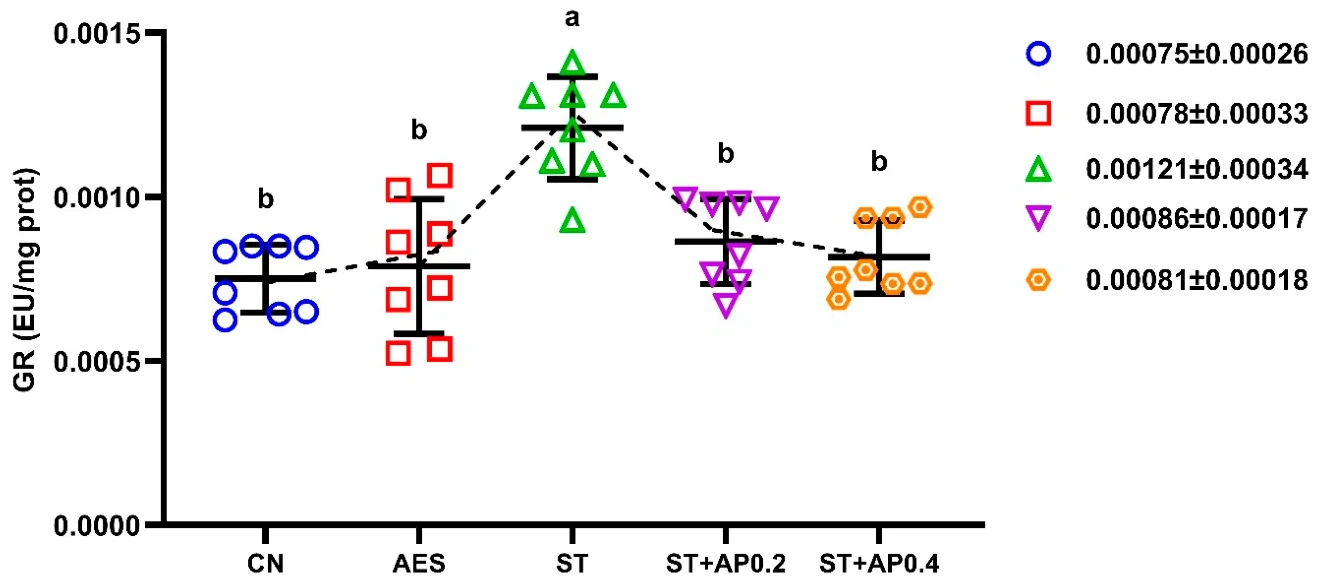
Erythrocyte GR activity across experimental groups. Data are presented as mean ± SD (n = 8 per group). Groups sharing the same letter are not significantly different, whereas groups with different letters differ significantly at *p* < 0.05 according to one-way ANOVA followed by Tukey’s multiple comparisons test. GR, glutathione reductase. CN, control; AES, acute exhaustive swimming; ST, short-term repeated swimming (vehicle-treated); ST + AP0.2 and ST + AP0.4, short-term repeated swimming plus apilarnil at 0.2 and 0.4 g·kg^−1^·day^−1^, respectively.

GR activity differed significantly among groups (F(4, 35) = 12.99, *p* < 0.001, η^2^ = 0.60, ω^2^ = 0.55). Erythrocyte GR activity did not differ significantly between the CN and AES groups (*p* = 0.99). GR activity was significantly higher in the ST group than in both CN and AES groups (both *p* < 0.001). Compared with ST, GR activity was significantly lower in both the ST + AP0.2 group (mean difference 3.46 × 10^−4^ EU/mg protein, 95% CI 1.37 × 10^−4^ to 5.56 × 10^−4^, *p* < 0.001) and the ST + AP0.4 group (mean difference 3.94 × 10^−4^ EU/mg protein, 95% CI 1.84 × 10^−4^ to 6.03 × 10^−4^, *p* < 0.001). GR activity in the ST + AP0.2 and ST + AP0.4 groups did not differ significantly from CN or AES (*p* > 0.05); however, this lack of a significant difference should not be interpreted as equivalence to control values. No significant difference was observed between the two apilarnil doses (*p* = 0.97) (Figure 4).

### 3.5. Erythrocyte TrxR Activity

**Figure 5:**
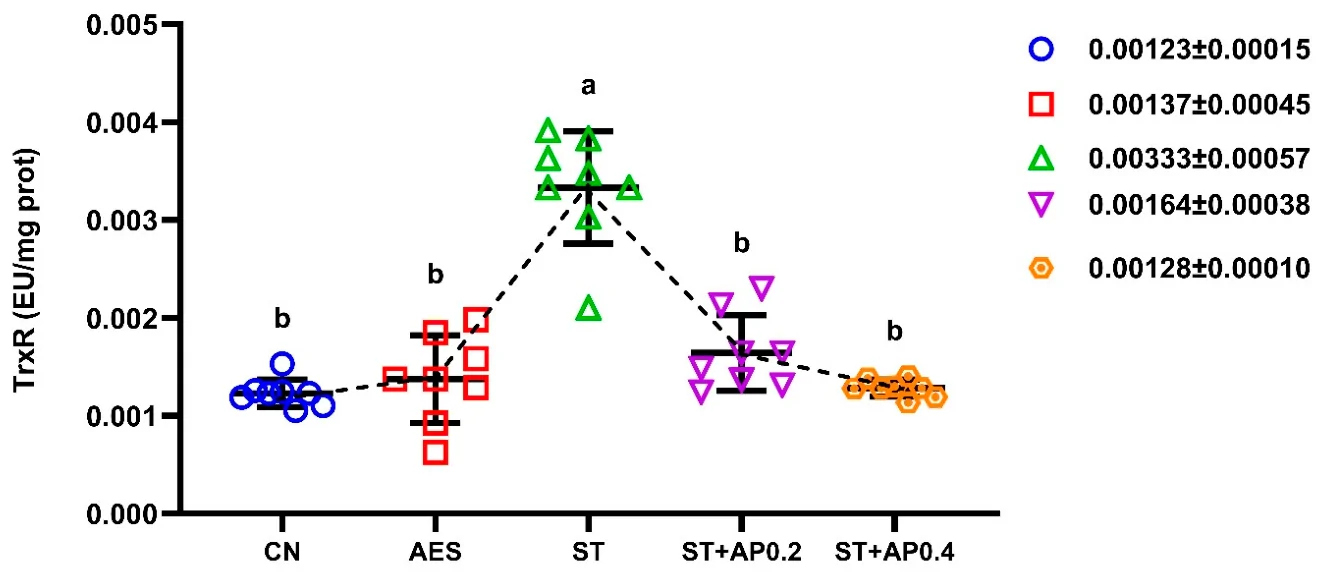
Erythrocyte TrxR activity across experimental groups. Data are presented as mean ± SD (n = 8 per group). Groups sharing the same letter are not significantly different, whereas groups with different letters differ significantly at *p* < 0.05 according to Welch’s ANOVA followed by Dunnett’s T3 multiple comparisons test. TrxR, thioredoxin reductase. CN, control; AES, acute exhaustive swimming; ST, short-term repeated swimming (vehicle-treated); ST + AP0.2 and ST + AP0.4, short-term repeated swimming plus apilarnil at 0.2 and 0.4 g·kg^−1^·day^−1^, respectively.

TrxR activity differed significantly among groups (Welch’s ANOVA: W(4.00, 15.94) = 24.14, *p* < 0.001; η^2^ = 0.84). Erythrocyte TrxR activity did not differ significantly between the CN and AES groups (*p* = 0.98). TrxR activity was significantly higher in the ST group than in both CN and AES groups (both *p* < 0.001). Compared with ST, TrxR activity was significantly lower in both the ST + AP0.2 group (mean difference 1.69 × 10^−3^ EU/mg protein, 95% CI 0.87 × 10^−3^ to 2.51 × 10^−3^, *p* < 0.001) and the ST + AP0.4 group (mean difference 2.05 × 10^−3^ EU/mg protein, 95% CI 1.27 × 10^−3^ to 2.83 × 10^−3^, *p* < 0.001). TrxR activity in the ST + AP0.2 and ST + AP0.4 groups did not differ significantly from CN or AES (*p* > 0.05); however, this lack of a significant difference should not be interpreted as equivalence to control values. No significant difference was observed between the two apilarnil doses (*p* = 0.22) (Figure 5).

### 3.6. Overall Pattern of Enzyme Activity Responses

Overall, a single terminal acute exhaustive swimming challenge did not significantly alter the measured erythrocyte enzyme activities compared with basal sedentary controls (*p* > 0.05). Repeated swimming exposure followed by the same terminal exhaustive challenge was associated with significantly higher G6PD, 6PGD, GST, GR, and TrxR activities (*p* < 0.05). Apilarnil supplementation was associated with differences in selected enzyme activities. The 0.4 g·kg^−1^·day^−1^ dose was associated with significantly lower GST activity than ST (*p* < 0.05), whereas both apilarnil doses were associated with significantly lower GR and TrxR activities (*p* < 0.05). In contrast, neither G6PD nor 6PGD activity differed significantly among the ST, ST + AP0.2, and ST + AP0.4 groups (*p* > 0.05).

### 3.7. Limitations of the Study

This study has several limitations. First, an apilarnil-only sedentary group was not included; therefore, the independent effect of apilarnil on basal erythrocyte enzyme activities could not be determined. Accordingly, the observed effects of apilarnil should be interpreted specifically within the context of repeated swimming exposure followed by the terminal exhaustive challenge. Consequently, lower GR, TrxR, or GST activities in the supplemented groups may represent either an exercise × apilarnil interaction or an effect of apilarnil independent of exercise.

Second, the study was limited to erythrocyte enzyme activity measurements. Direct oxidative damage markers, ROS production, glutathione redox status, erythrocyte hemolysis resistance, and upstream molecular pathways were not assessed. Therefore, the findings should be interpreted as evidence of enzyme activity modulation rather than definitive evidence of reduced oxidative stress, antioxidant protection, or specific molecular pathway activation.

Third, exercise intensity during the repeated swimming sessions was not individually quantified using physiological measures such as blood lactate, indirect calorimetry, or externally applied workload. In addition, the 14-day intervention period was relatively short compared with conventional chronic aerobic training protocols. Therefore, the exercise protocol should be interpreted as short-term repeated swimming exposure rather than a precisely quantified long-term aerobic training stimulus.

Fourth, and most importantly, all repeatedly exercised groups underwent a terminal exhaustive swimming challenge 1 h before blood collection, and no repeatedly exercised group was sampled without this challenge. This is a fundamental feature of the design rather than a minor limitation: the higher enzyme activities observed in the ST group cannot be attributed specifically to the preceding 14-day exposure, and a repeatedly exercised group sampled without the terminal test would be required to isolate chronic adaptation. 

Fifth, swimming time to exhaustion during the terminal challenge was not recorded. Consequently, it cannot be excluded that the apilarnil-treated animals swam for a different duration than vehicle-treated animals, and differences in enzyme activities may partly reflect differences in the duration or intensity of the terminal exercise stimulus rather than a direct effect of apilarnil. In addition, blood was sampled 1 h after the terminal challenge; therefore, the measured enzyme activities reflect the early recovery period rather than the immediate post-exercise state. Because erythrocyte GR and GST activities have been shown to change in a time-dependent manner after strenuous exercise [34], the magnitude and direction of group differences might differ at other sampling times. 

Sixth, because younger erythrocytes and reticulocytes exhibit higher activities of several enzymes, including G6PD, exercise-related shifts in erythrocyte age distribution or in the proportion of non-hemoglobin proteins could influence protein-normalized activities. This possibility cannot be excluded, because hemoglobin concentration, hematocrit, and other hematological indices were not measured. Likewise, complete blood counts, including erythrocyte indices such as mean corpuscular volume (MCV) and mean corpuscular hemoglobin concentration (MCHC), markers of hemolysis, and basic biochemical parameters such as ALT, AST, and plasma total protein were not assessed. Therefore, exercise- or apilarnil-related changes in erythrocyte size and hemoglobin content, hemoconcentration, plasma volume shifts, hemolysis, or hepatic function could not be evaluated. Inclusion of these parameters in future studies would help to interpret erythrocyte enzyme activities in relation to erythrocyte characteristics and systemic safety.

Finally, enzyme activities were normalized to total hemolysate protein rather than hemoglobin concentration, and measurements were obtained at a single post-exercise time point. Only male rats were included. Because sex may influence exercise responses, erythrocyte redox metabolism, and responses to dietary supplementation, replication in female animals is warranted before these findings can be generalized. Future studies should include apilarnil-only control groups, direct redox and oxidative damage markers, standardized exercise intensity, multiple sampling time points, broader dose ranges, and functional performance outcomes. 

## 4. Discussion

The present study investigated whether apilarnil supplementation modifies erythrocyte NADPH-dependent and thiol-related enzyme activities in rats subjected to short-term repeated swimming and a terminal acute exhaustive swimming challenge. The main findings were that a single terminal acute exhaustive swimming challenge did not significantly alter the measured erythrocyte enzyme activities compared with basal sedentary controls, whereas repeated swimming exposure followed by the same terminal challenge was associated with higher G6PD, 6PGD, GST, GR, and TrxR activities. Apilarnil supplementation was associated with differences in selected enzyme activities. The higher dose was associated with lower GST activity compared with ST, while both doses were associated with lower GR and TrxR activities. In contrast, neither G6PD nor 6PGD activity was significantly affected by apilarnil supplementation.

The absence of significant differences between the CN and AES groups suggests that a single terminal acute exhaustive swimming challenge was not sufficient to alter erythrocyte enzyme activity under the present experimental conditions. This finding does not exclude the possibility of transient acute redox changes after exhaustive exercise; however, mature erythrocytes have limited capacity for rapid enzymatic remodeling because they lack nuclei and mitochondria. In this context, the AES group provided a reference for comparing the enzymatic response to an isolated acute challenge with that observed after repeated swimming exposure followed by the same terminal challenge. This interpretation is broadly consistent with previous studies indicating that the erythrocyte response to exhaustive exercise depends on prior training status. In rats, endurance training altered the erythrocyte superoxide dismutase and glutathione peroxidase responses to acute exhaustive exercise and prevented exercise-induced increases in erythrocyte malondialdehyde [35]. Similarly, one month of regular treadmill training prevented exhaustive-exercise-induced lipid peroxidation and structural deterioration of erythrocytes observed in sedentary rats [36]. In humans, erythrocyte GR and GST activities increased progressively over several months of running training [36]. However, these studies used longer training periods, different exercise modalities, and partly different enzymes; therefore, they support the concept of training-dependent erythrocyte redox responses rather than providing direct comparisons with the present findings. In addition, because blood was sampled 1 h after the challenge, transient changes occurring immediately after exhaustive swimming may have resolved by the time of sampling.

Repeated swimming exposure followed by the terminal exhaustive challenge was associated with higher activities of both PPP-related and thiol-related enzymes. The higher G6PD and 6PGD activities are consistent with increased activity of NADPH-generating enzymes within the pentose phosphate pathway. This is biologically relevant because NADPH is required for glutathione and thioredoxin recycling, both of which are central to erythrocyte redox homeostasis. The simultaneous increases in GST, GR, and TrxR further indicate changes in glutathione- and thioredoxin-related enzyme activities. This pattern would be compatible with the concept of exercise-induced redox hormesis [3,4,5,37,38]. However, because all repeatedly exercised animals were sampled 1 h after a terminal exhaustive challenge and no repeatedly exercised group was sampled without this challenge, the present data cannot be regarded as direct evidence of training-induced antioxidant adaptation. The higher activities in the ST group may reflect the repeated swimming exposure, the response of a previously exercised organism to the terminal challenge, or their interaction.

Apilarnil supplementation did not significantly alter the activities of the PPP-related enzymes. Although mean G6PD activity was numerically lower in the apilarnil-treated groups, particularly at the higher dose, this difference did not reach statistical significance, and 6PGD activity was similar among the ST and apilarnil-treated groups. Thus, under the present conditions, apilarnil was not associated with significant changes in erythrocyte NADPH-generating enzyme activities. Because NADPH/NADP+ balance was not measured, the present findings do not allow conclusions regarding NADPH availability.

Apilarnil supplementation was also associated with differences in thiol-related enzyme activities. GR and TrxR activities were significantly lower in both apilarnil-treated groups compared with the ST group, whereas GST activity was significantly lower only at the higher dose. However, lower enzyme activity should not be interpreted as inherently beneficial. Lower GR, TrxR, or GST activity may reflect a reduced oxidative challenge or enzymatic requirement, but it may equally reflect direct enzyme inhibition by constituents of the apilarnil matrix, altered substrate or cofactor availability, or changes in the erythrocyte population. Because ROS, GSH/GSSG, NADPH/NADP+, lipid peroxidation, protein oxidation, and hemolysis were not measured, these possibilities cannot be distinguished. Therefore, the observed differences are more appropriately interpreted as evidence of erythrocyte enzyme activity modulation.

The effects of apilarnil differed among the measured enzymes and between the two administered doses. GST activity was significantly lower than in the ST group only at the higher dose, whereas GR and TrxR activities were significantly lower at both doses, and neither PPP-related enzyme was significantly affected. These findings indicate that apilarnil was associated predominantly with differences in thiol-related rather than NADPH-generating enzyme activities. Because no sedentary apilarnil-treated groups were included, it cannot be determined whether these differences reflect an interaction between apilarnil and repeated swimming or an independent effect of apilarnil on erythrocyte enzyme activity. All apilarnil-related findings therefore refer strictly to differences observed within the repeated-swimming context.

The doses used in the present study (0.2 and 0.4 g·kg^−1^·day^−1^) correspond to the lowest and intermediate doses of the 0.2–0.8 g·kg^−1^ range previously tested in rat models of lipopolysaccharide-induced liver and kidney injury, in which apilarnil exerted antioxidant and anti-inflammatory effects [21,22]. Within the currently available experimental literature, they can therefore be regarded as low-to-moderate exposures, and the highest previously tested dose was not included. However, because dose–response, pharmacokinetic, and toxicity data for apilarnil in exercising animals are lacking, and because the previous studies used pathological rather than exercise models, this classification is relative rather than absolute. Whether higher doses would produce larger or qualitatively different effects on erythrocyte enzyme activities remains to be determined. No extrapolation to human sports nutrition is intended.

The biological activity of apilarnil may be related to its complex chemical composition. The apilarnil product used in this study contained several phenolic acids and flavonoids [23]. Phenolic acids and flavonoids have been associated with antioxidant, anti-inflammatory, and redox-related effects in experimental models [19]. Previous studies have also reported antioxidant and anti-inflammatory effects of apilarnil in experimental injury models, including modulation of TLR4/NF-κB-related pathways [21,22]. Some of these constituents have been reported to interact directly with the enzymes measured in the present study. In vitro, caffeic acid inhibited G6PD and 6PGD purified from human erythrocytes, whereas p-coumaric acid had no inhibitory effect on either enzyme [39]. Quercetin has been shown to inhibit mammalian TrxR [40]. Such interactions could theoretically contribute to the numerically lower G6PD activity and the significantly lower TrxR activity observed in the apilarnil-treated groups. However, these effects were demonstrated at concentrations and under conditions that may not be reached in circulating erythrocytes after oral administration, and the relative abundance of these constituents in apilarnil, their bioavailability, and their erythrocyte exposure were not determined. Therefore, this possibility should be regarded as a hypothesis for future studies rather than an explanation of the present findings. Therefore, the observed effects should be interpreted as responses to the apilarnil matrix as a whole rather than effects attributable to any specific constituent.

The timing of supplementation should also be considered. In the present study, apilarnil was administered throughout the 14-day repeated swimming period rather than as a single acute intervention. Therefore, the present findings should be interpreted specifically in the context of repeated apilarnil administration accompanying repeated exercise exposure and cannot be extrapolated to acute pre- or post-exercise supplementation.

From a translational perspective, these findings provide preliminary experimental evidence that apilarnil supplementation may modify erythrocyte enzyme activity responses associated with repeated exercise exposure. However, the results should not be directly extrapolated to athletes, clinical populations, or sports nutrition practice. Future studies should include direct oxidative stress and damage markers, hemolysis-related outcomes, functional performance measures, and a broader range of apilarnil doses before practical recommendations can be made.

## 5. Conclusions

This study showed that repeated swimming exposure followed by a terminal exhaustive swimming challenge was associated with higher erythrocyte G6PD, 6PGD, GST, GR, and TrxR activities in rats, whereas a single acute exhaustive swimming challenge alone did not significantly alter these enzyme activities. Apilarnil supplementation was associated with differences in selected erythrocyte enzyme activities. The higher apilarnil dose was associated with lower GST activity compared with the ST group, whereas both doses were associated with lower GR and TrxR activities; G6PD and 6PGD activities were not significantly affected by apilarnil supplementation. Because direct oxidative stress markers, glutathione redox status, hemolysis resistance, and molecular pathways were not assessed, these findings should be interpreted as preliminary evidence of erythrocyte enzyme activity modulation rather than definitive evidence of oxidative protection or enhanced exercise adaptation.

## Figures and Tables

**Table 1:** Summary of the experimental protocol.

Experimental Phase	Procedure	Details
Animals	Experimental animals	Forty adult male Wistar rats randomly allocated into five groups (n = 8/group).
Acclimation	Water acclimation	7 days (AES, ST, ST + AP0.2 and ST + AP0.4 groups only).
Intervention	Repeated swimming	30 min/day for 14 consecutive days (ST, ST + AP0.2 and ST + AP0.4 groups).
Supplementation	Oral gavage	AES and ST: physiological saline (0.5 mL/day); ST + AP0.2: apilarnil 0.2 g·kg^−1^·day^−1^; ST + AP0.4: apilarnil 0.4 g·kg^−1^·day^−1^.
Terminal challenge	Exhaustive swimming	Performed on Day 15 in AES, ST, ST + AP0.2 and ST + AP0.4 groups.
Blood sampling	Sample collection	Blood collected from the abdominal vein into EDTA tubes under ketamine/xylazine anesthesia.
Sample preparation	Erythrocyte isolation	Hemolysate preparation and storage at −80 °C until analysis.
Biochemical analyses	Spectrophotometric assays	G6PD, 6PGD, GST, GR and, TrxR activities; Bradford protein assay.

AES: Acute exhaustive swimming; ST: Short-term repeated swimming (vehicle-treated); AP: Apilarnil; G6PD: Glucose-6-phosphate dehydrogenase; 6PGD: 6-phosphogluconate dehydrogenase; GST: glutathione S-transferase; GR: Glutathione reductase; TrxR: Thioredoxin reductase.

## Data Availability

The datasets generated and/or analyzed during the current study are available from the corresponding author upon reasonable request.

## Abbreviations

AES	Acute exhaustive swimming
AP	Apilarnil
ARRIVE	Animal Research: Reporting of In Vivo Experiments
BSA	Bovine Serum Albumin
CN	Control group
DTNB	5,5′-Dithiobis-(2-nitrobenzoic acid)
EU	Enzyme Unit
G6PD	Glucose-6-Phosphate Dehydrogenase
6PGD	6-Phosphogluconate Dehydrogenase
GR	Glutathione Reductase
GST	Glutathione S-Transferase
LC-MS/MS	Liquid Chromatography-Tandem Mass Spectrometry
NADPH	Nicotinamide Adenine Dinucleotide Phosphate
PPP	Pentose Phosphate Pathway
ROS	Reactive Oxygen Species
SD	Standard Deviation
ST	Short-term repeated swimming (vehicle-treated)
TrxR	Thioredoxin Reductase
